# Uterine Fibroids in Pregnancy: Maternal, Obstetric, and Neonatal Outcomes in a Retrospective Cohort Study

**DOI:** 10.3390/diagnostics16203358

**Published:** 2026-10-15

**Authors:** Francesco Giuseppe Martire, Claudia d’Abate, Eugenia Costantini, Giulia Gavanelli, Ilaria Ianes, Maria De Bonis, Giuseppe Sorrenti, Errico Zupi, Lucia Lazzeri

**Affiliations:** 1 Department of Molecular and Developmental Medicine, Obstetrics and Gynecological Clinic, University of Siena, 53100 Siena, Italy; francescogmartire@libero.it (F.G.M.);; 2 Department of Gynecology, San Carlo Nancy Hospital, 00165 Rome, Italy

**Keywords:** fibroids, pregnancy, obstetric complications, neonatal outcomes

## Abstract

**Background**: Uterine fibroids are the most common benign tumors in women of reproductive age, and their coexistence with pregnancy is becoming increasingly frequent as maternal age at first pregnancy rises. **Objective**: The objective of this study was to evaluate the impact of uterine fibroids on obstetric and neonatal outcomes and, secondarily, the effect of pregnancy on fibroid growth. **Study Design**: This retrospective observational study included 1512 pregnant women, 136 of whom had uterine fibroids, who were followed at a university hospital. Ultrasound examinations were performed before pregnancy (T0) and during the first (T1), second (T2), and third (T3) trimesters. Fibroid number, size, location, and ultrasound characteristics according to FIGO classification were recorded. Obstetric complications, mode of delivery, and neonatal outcomes were analyzed according to fibroid type and size. Fibroid growth throughout pregnancy was also evaluated. **Results**: Among 1512 pregnant women followed between 1 January 2024 and 30 November 2025, 136 patients (9.0%) had uterine fibroids. A positive family history of fibroids was identified in 36% of cases. Gestational complications were infrequent overall, although gestational hypothyroidism was significantly more common among women with fibroids, whereas preeclampsia and postpartum hemorrhage were significantly less frequent in univariable analysis. Vaginal delivery was less frequent in the fibroid group, while operative vaginal delivery and cesarean section were significantly more common. Fetal growth restriction (FGR) and umbilical venous pH also differed significantly between groups. In multivariable analysis adjusted for maternal age, BMI, parity, ART, smoking, chronic hypertension, and diabetes, the association with cesarean section was attenuated (aOR 1.47, 95% CI 0.93–2.32), whereas lower PPH rates persisted (aOR 0.34, 95% CI 0.14–0.84) and a slight inverse association with umbilical venous pH remained detectable (*β* −0.01, *p* = 0.01). Exploratory stratification according to FIGO classification (3–4 vs. 5–7) showed significant differences in PPH, cesarean section, FGR, and LGA. Repeated-measure analysis demonstrated progressive fibroid growth in maximum diameter during pregnancy compared with preconceptional measurements (T0) (*p* < 0.001), with substantial enlargement already observed by the second trimester and fibroids ≤5 cm exhibiting greater relative growth at T2 compared with T0 than larger lesions (+18% vs. +7%; *p* = 0.03). **Conclusions**: Uterine fibroids were not consistently associated with adverse obstetric or neonatal outcomes, although careful obstetric surveillance remains warranted. Pregnancy was associated with progressive fibroid growth, particularly in smaller lesions and during early gestation.

## 1. Introduction

Leiomyomas are the most prevalent benign solid tumors of the uterus and grow mainly under estrogen stimulation; however, their pathogenesis and risk factors remain incompletely understood [1,2,3,4].

Most fibroids are diagnosed incidentally—found in 30–50% of asymptomatic premenopausal women—while 25–30% develop symptoms like abnormal uterine bleeding (AUB), pelvic pain, pressure-related symptoms, anemia, or infertility [5]. In particular, submucosal and intramural fibroids can impair fertility by altering endometrial receptivity, distorting the uterine cavity, disrupting myometrial contractility and blood flow, and inducing chronic inflammation or microbiome alterations [6].

Uterine fibroids are detected in 1–10% of pregnancies [7,8]. As childbearing is increasingly delayed, their incidence during pregnancy is expected to rise [9,10,11]. Since fibroids have been associated with adverse obstetric outcomes, their identification, characterization, and monitoring during pregnancy are clinically relevant [12,13].

From a clinical perspective, early evaluation should document the number, maximum diameter, FIGO type, and relationship of the dominant lesion with the endometrial cavity, placenta, cervix, and lower uterine segment. Because fibroid behavior in pregnancy is unpredictable, serial ultrasound assessments track growth and vascular changes, enabling personalized surveillance tailored to patient symptoms and obstetric findings [7].

Gestational fibroid growth is highly variable: approximately 60% of fibroids may increase or decrease by more than 10% of their original size [14]. Hammoud et al. reported that during the first half of pregnancy, 44.9% of fibroids increased in volume, whereas 55.1% decreased; later in pregnancy, 75% of fibroids decreased in size [15]. Other studies have similarly described rapid enlargement in early gestation and different second-trimester growth patterns according to baseline fibroid size [15,16,17]. Growth may reflect hormonal stimulation and increased angiogenesis, whereas uterine expansion, ischemic changes, and postpartum uterine contractions may contribute to regression [15].

Approximately 10–30% of pregnant women with fibroids develop complications, including spontaneous abortion, preterm labor and preterm birth, placental abruption, placenta previa, malpresentation, labor dystocia, cesarean delivery, and postpartum hemorrhage (PPH) [3,9,11].

However, the mechanisms underlying these associations remain unclear. Therefore, this study aimed primarily to evaluate the association between uterine fibroids and obstetric and neonatal outcomes, including the assessment of potential differences after adjustment for relevant maternal and pregnancy-related confounders. The secondary aims of this study were to investigate whether fibroid characteristics, including FIGO type and baseline size, are associated with adverse obstetric and neonatal outcomes, and to assess the effect of pregnancy on fibroid growth according to fibroid location and baseline size. These findings could help, first and foremost, to provide counseling to these patients regarding their underlying condition and, subsequently, to determine how to tailor their care before, during, and after pregnancy.

## 2. Materials and Methods

This retrospective observational study was conducted through a review of the medical records of pregnant women followed at the Department of Obstetrics and Gynecology of Siena University Hospital between 1 January 2024 and 30 November 2025. A total of 1512 patients were included. This study included pregnant women attending the prenatal outpatient clinic of our institution who subsequently delivered at our center. The inclusion criteria were the presence of one documented baseline ultrasound examination performed at our center within 12 months prior to conception; antenatal care and delivery conducted entirely at our department; complete clinical, obstetric, and sonographic documentation available; and signed informed consent.

The exclusion criteria were the presence of major fetal congenital anomalies; pre-existing absolute contraindications to vaginal delivery unrelated to fibroid pathology; and incomplete follow-up or missing perinatal outcome data. Isolated minor congenital anomalies were not considered exclusion criteria.

For each case, medical history, clinical, obstetric, neonatal, and ultrasound data were collected, including pre-existing conditions, pregnancy-related complications, maternal–fetal outcomes, and the presence and ultrasound characteristics of uterine fibroids. This study was conducted in accordance with the 2013 Declaration of Helsinki and was approved by the local Ethics Committee (protocol code: PEOPLE; protocol number: 29792).

Baseline variables included maternal age, ethnicity, pre-pregnancy body mass index (BMI), gestational weight gain, smoking status, alcohol consumption, family history of uterine fibroids, chronic hypertension, diabetes mellitus, thyroid dysfunction, age at menarche, previous gynecological disorders including endometriosis, adenomyosis, and PMOS, and obstetric history, including parity, number of pregnancies, and mode of conception, either spontaneous or through assisted reproductive technologies (ARTs). Obstetric complications included gestational hypertension, preeclampsia/eclampsia, intrahepatic cholestasis of pregnancy, gestational diabetes mellitus (GDM), placental abnormalities, threatened preterm labor, postpartum hemorrhage, and labor abnormalities. Mode of delivery was classified as vaginal delivery, operative vaginal delivery, or cesarean section, and vaginal or perineal lacerations were graded from the first to fourth degree. Fetal growth abnormalities included fetal growth restriction (FGR), small for gestational age (SGA), and large for gestational age (LGA). Neonatal outcomes included congenital malformations, umbilical venous pH at delivery, gestational age at birth, birth weight, and neonatal sex. Umbilical venous blood was sampled immediately after delivery from the clamped umbilical cord and analyzed promptly using a blood gas analyzer according to the routine protocol of our institution. Venous pH values recorded in the clinical charts were used for the present analysis.

### 2.1. Outcome Definitions

Postpartum hemorrhage (PPH) was defined as blood loss ≥ 500 mL following vaginal delivery or ≥1000 mL following cesarean section. Blood loss was quantitatively measured using graduated collection drapes during vaginal deliveries and graduated suction canisters during cesarean sections, after subtraction of amniotic fluid and irrigation fluids.

Preeclampsia was defined as new-onset hypertension (systolic blood pressure ≥ 140 mmHg and/or diastolic blood pressure ≥ 90 mmHg) after 20 weeks of gestation, associated with proteinuria (≥300 mg/24 h), maternal organ dysfunction, or uteroplacental dysfunction, including fetal growth restriction, according to ISSHP diagnostic criteria [18].

Fetal growth abnormalities were diagnosed prenatally using Hadlock reference charts [19]. Small for gestational age (SGA) was defined as an estimated fetal weight between the 3rd and 10th percentiles with normal Doppler findings, whereas fetal growth restriction (FGR) was diagnosed according to the Delphi consensus criteria, incorporating fetal biometry, growth velocity, and Doppler abnormalities according to gestational age [20,21]. SGA and FGR were recorded as mutually exclusive diagnostic categories. Large for gestational age (LGA) was defined as an estimated fetal weight above the 90th percentile.

Gestational hypothyroidism included both overt and subclinical forms, diagnosed according to pregnancy-specific TSH and free thyroxine (FT4) reference ranges, in collaboration with the Endocrinology Unit of our institution [22].

### 2.2. Ultrasound Assessment

All sonographic examinations were performed by two experienced maternal–fetal medicine specialists using high-resolution ultrasound systems (Voluson E10 and Voluson S10; GE Healthcare, Chicago, IL, USA) equipped with convex transabdominal (3.0–5.0 MHz) and transvaginal (5.0–9.0 MHz) transducers. Measurements were performed according to the same standardized protocol at all study timepoints.

To ensure accurate longitudinal evaluation of fibroid dynamics, eligibility was restricted to patients with complete sonographic documentation available across four standardized timepoints: a preconceptional baseline scan performed within the 12 months preceding pregnancy (*T*_0_), followed by gestational assessments in the first (*T*_1_, 11^+0^–13^+6^ weeks), second (*T*_2_, 19^+0^–21^+6^ weeks), and third (*T*_3_, 30^+0^–34^+6^ weeks) trimesters. Specifically, preconceptional scans (*T*_0_) were included only if they explicitly documented complete baseline parameters, including fibroid number, maximum diameter, anatomical location, and baseline vascularization. Fibroid location was classified according to the FIGO 2018 system [23]. In patients with multiple fibroids, the largest lesion identified at the preconceptional assessment (T0) was selected as the index fibroid and consistently followed at all subsequent ultrasound examinations (T1, T2, and T3). Longitudinal growth was assessed using the maximum fibroid diameter, expressed in millimeters (mm).

Subsequent gestational records (*T*_1_–*T*_3_) were then analyzed to evaluate interval changes in fibroid dimensions, internal echogenicity, and spatial relationships with the endometrial cavity, placenta, cervix, and lower uterine segment.

Changes in maximum fibroid diameter throughout pregnancy, together with obstetric parameters including fetal growth, placental location, and amniotic fluid volume, were monitored to identify potentially related complications.

### 2.3. Statistical Analysis

Descriptive analyses were performed for the whole cohort. Continuous variables were reported as mean ± standard deviation or median and interquartile range, according to data distribution; categorical variables were expressed as frequencies and percentages. Comparisons between women with and without uterine fibroids were performed to assess differences in baseline characteristics, obstetric complications, and neonatal outcomes. Categorical variables were compared using the chi-square test or Fisher’s exact test, as appropriate, while continuous variables were compared using Student’s *t*-test or the Mann–Whitney U test according to data distribution.

Multivariable regression analyses were performed for selected clinically relevant obstetric and neonatal outcomes to account for potential confounding. Multivariable logistic regression models were used for binary outcomes, and linear regression models were used for continuous outcomes. The primary adjustment set included maternal age, body mass index (BMI), parity, use of assisted reproductive technology (ART), smoking status, chronic hypertension, and pre-existing diabetes mellitus or gestational diabetes mellitus. Adjusted odds ratios (ORs), with corresponding 95% confidence intervals (CIs), were reported.

For outcomes characterized by rare events or complete/quasi-complete separation, penalized logistic regression was used when appropriate. The number of covariates included in each model was considered in relation to the number of observed outcome events to minimize the risk of model overfitting. Outcomes with an insufficient number of events for reliable multivariable modeling were not subjected to adjusted analysis.

Exploratory subgroup analyses assessed whether fibroid type (FIGO types 3–4 vs. FIGO types 5–7) and size (≤5 cm vs. >5 cm) were associated with adverse obstetric and neonatal outcomes. Given the number of outcomes and subgroup comparisons, these analyses were considered hypothesis-generating and were interpreted with emphasis on effect estimates and 95% confidence intervals, where available, rather than on nominal p values alone. For fibroid-specific analyses, longitudinal changes in maximum fibroid diameter (mm) were assessed using linear mixed-effect models (LMMs). Timepoint (T0, T1, T2, and T3), fibroid subgroup (FIGO types 3–4 vs. 5–7 or baseline diameter ≤ 5 cm vs. >5 cm), and the timepoint × subgroup interaction were included as fixed effects. A patient-level random intercept was used to account for repeated measurements within individuals. Post hoc pairwise comparisons were adjusted using Bonferroni’s correction. No volume transformation was performed, as fibroid diameter was the dependent variable. Relative changes in maximum fibroid diameter were calculated using the preconceptional measurement (T0) as the reference.

## 3. Results

This study included 1512 pregnant women followed at the Department of Obstetrics and Gynecology of Siena University Hospital. Among them, 136 patients (9.0%) had an ultrasound diagnosis of uterine fibroids, whereas 1376 (91.0%) showed no evidence of fibroids. Regarding demographic and general clinical characteristics, maternal age did not differ significantly between groups (34 ± 8.9 vs. 35 ± 8.8 years; *p* = 0.21). In contrast, women with fibroids had a significantly lower pre-pregnancy BMI (24.4 ± 6.0 vs. 25.9 ± 5.0 kg/m^2^; *p* = 0.002) and lower gestational weight gain (14 ± 3.4 vs. 15 ± 4.9 kg; *p* = 0.02). Caucasian ethnicity was significantly more common among women with fibroids than among controls (92.6% vs. 85.3%; *p* = 0.02). Smoking and alcohol consumption were significantly more frequent among women with fibroids than among controls (26.4% vs. 14.0%, *p* < 0.001; and 20.6% vs. 8.0%, *p* < 0.001, respectively). Among pre-existing medical conditions, thyroid disorders (15.4% vs. 8.2%; *p* = 0.01) and chronic hypertension (3.7% vs. 1.3%; *p* = 0.04) were significantly more frequent among women with fibroids, whereas the prevalence of type I and type II diabetes mellitus did not differ significantly between groups (Table 1).

Regarding gynecologic and reproductive characteristics, a positive family history of uterine fibroids was significantly more common in the fibroid group (36.0% vs. 20.0%; *p* < 0.001). Women with fibroids also had a significantly lower age at menarche (11 ± 1 vs. 13 ± 3 years; *p* < 0.001) and a higher prevalence of PMOS (11.0% vs. 5.6%; *p* = 0.01), whereas endometriosis/adenomyosis showed no significant association with leiomyomas. Mean parity and number of pregnancies were similar between groups. ART was significantly more frequent among women with fibroids (22.1% vs. 7.3%; *p* < 0.001), whereas spontaneous conception was less common (77.9% vs. 92.7%; *p* < 0.001). Singleton and twin pregnancies were similarly distributed between groups (Table 2).

As shown in Table 3, gestational complications were infrequent overall. The rates of gestational hypertension, cholestasis, GDM, placental abnormalities, threatened preterm labor, and labor abnormalities did not significantly differ between groups (*p* > 0.05). In contrast, preeclampsia was significantly less frequent among women with fibroids (0% vs. 8.8%; OR 0.04, 95% CI 0.00–0.61; *p* < 0.01). Gestational hypothyroidism was significantly more frequent in women with fibroids (20.6% vs. 6.1%; OR 3.99, 95% CI 2.49–6.38; *p* < 0.001), whereas postpartum hemorrhage was significantly less frequent in the fibroid group (3.7% vs. 12.1%; OR 0.28, 95% CI 0.11–0.68; *p* = 0.003).

Vaginal delivery was the most common mode of birth (62.1%) but occurred less frequently in women with fibroids than in controls (47.1% vs. 63.6%; OR 0.51, 95% CI 0.36–0.73; *p* < 0.001). Both operative vaginal delivery and cesarean section occurred more frequently in the fibroid group than in the control group, with statistically significant differences between groups (9.6% vs. 4.6%; OR 2.20, 95% CI 1.18–4.12; *p* = 0.01; and 43.4% vs. 31.8%; OR 1.64, 95% CI 1.15–2.35; *p* = 0.01, respectively). Labor induction was comparable between groups. Among women who delivered vaginally, second-degree perineal tears were significantly more frequent among women with fibroids than among controls (42.9% vs. 29.7%; OR 1.77, 95% CI 1.10–2.84; *p* = 0.02), whereas first-degree tears did not differ significantly between groups (40.3% vs. 38.3%; OR 1.09, 95% CI 0.68–1.75; *p* = 0.72). No significant differences were observed for third- or fourth-degree tears.

### Multivariable Analysis

In multivariable analyses (Table 4), the association between uterine fibroids and cesarean delivery was attenuated after adjustment for maternal age, BMI, parity, ART, smoking status, chronic hypertension, and diabetes mellitus/gestational diabetes while remaining in the same direction as in the univariable analysis (adjusted odds ratio [aOR], 1.47; 95% CI, 0.93–2.32; *p* = 0.10).

The inverse association observed between uterine fibroids and postpartum hemorrhage persisted after adjustment (aOR, 0.34; 95% CI, 0.14–0.84; *p* = 0.02). However, this finding should be interpreted cautiously given the small number of postpartum hemorrhage events in the fibroid group.

For intrauterine growth restriction, the adjusted analysis showed an increased point estimate in women with fibroids, although the confidence interval was wide and included the null value (aOR, 1.86; 95% CI, 0.86–4.03; *p* = 0.11).

No adjusted estimate could be reliably obtained for preeclampsia because no events occurred among women with uterine fibroids, resulting in complete separation in the logistic regression model.

In linear regression analyses, uterine fibroids were not associated with birthweight (β = +19.7 g; 95% CI −73.5 to +112.8; *p* = 0.68). A small inverse association was observed between fibroids and umbilical venous pH after adjustment (β, −0.01; 95% CI, −0.02 to −0.003; *p* = 0.01).

Regarding fetal and neonatal outcomes, FGR occurred in 58 cases (3.8%), including 10 cases in the fibroid group (7.4% vs. 3.5%; OR 2.20, 95% CI 1.08–4.45; *p* = 0.03). SGA neonates were identified in 11 cases (0.7%), all in the control group, with no statistically significant difference between groups. Similarly, the rate of LGA neonates did not differ significantly between women with and without fibroids (7.4% vs. 4.5%; OR 1.68, 95% CI 0.84–3.36; *p* = 0.14). No significant differences were observed in fetal malformations, gestational age at birth, birth weight, or fetal sex distribution. The 35 congenital anomalies recorded (2.3%) consisted exclusively of minor findings, including isolated single umbilical artery and mild renal pelvic dilatation. No major congenital anomalies were included in the study population.

Umbilical venous pH at delivery was significantly lower in the fibroid group (7.31 ± 0.05 vs. 7.32 ± 0.04; *p* = 0.01) (Table 5).

The 58 FGR cases and 11 SGA cases represented distinct, non-overlapping prenatal diagnostic categories, as defined in the Section 2.

Within the fibroid cohort, stratification according to FIGO classification (3–4 vs. 5–7) showed no significant differences in most obstetric and neonatal outcomes. However, postpartum hemorrhage was significantly more frequent among patients with FIGO type 3–4 fibroids (9.9% vs. 0%; *p* = 0.01). Among women who delivered vaginally, first-degree tears were significantly less frequent in patients with FIGO type 3–4 fibroids than in those with FIGO type 5–7 fibroids (21.7% vs. 48.1%; OR 0.30, 95% CI 0.10–0.92; *p* = 0.04), whereas second-degree tears did not differ significantly between groups (56.5% vs. 37.0%; OR 2.21, 95% CI 0.82–5.96; *p* = 0.14). Cesarean section and FGR were significantly more frequent among patients with FIGO type 3–4 fibroids (55.0% vs. 36.5%, *p* = 0.04; and 13.7% vs. 3.5%, *p* = 0.03, respectively), whereas LGA was significantly more frequent among patients with FIGO type 5–7 fibroids (11.8% vs. 0%; *p* = 0.01) (Table 6). These subgroup findings should be interpreted as exploratory given the number of comparisons performed.

**Figure 1:**
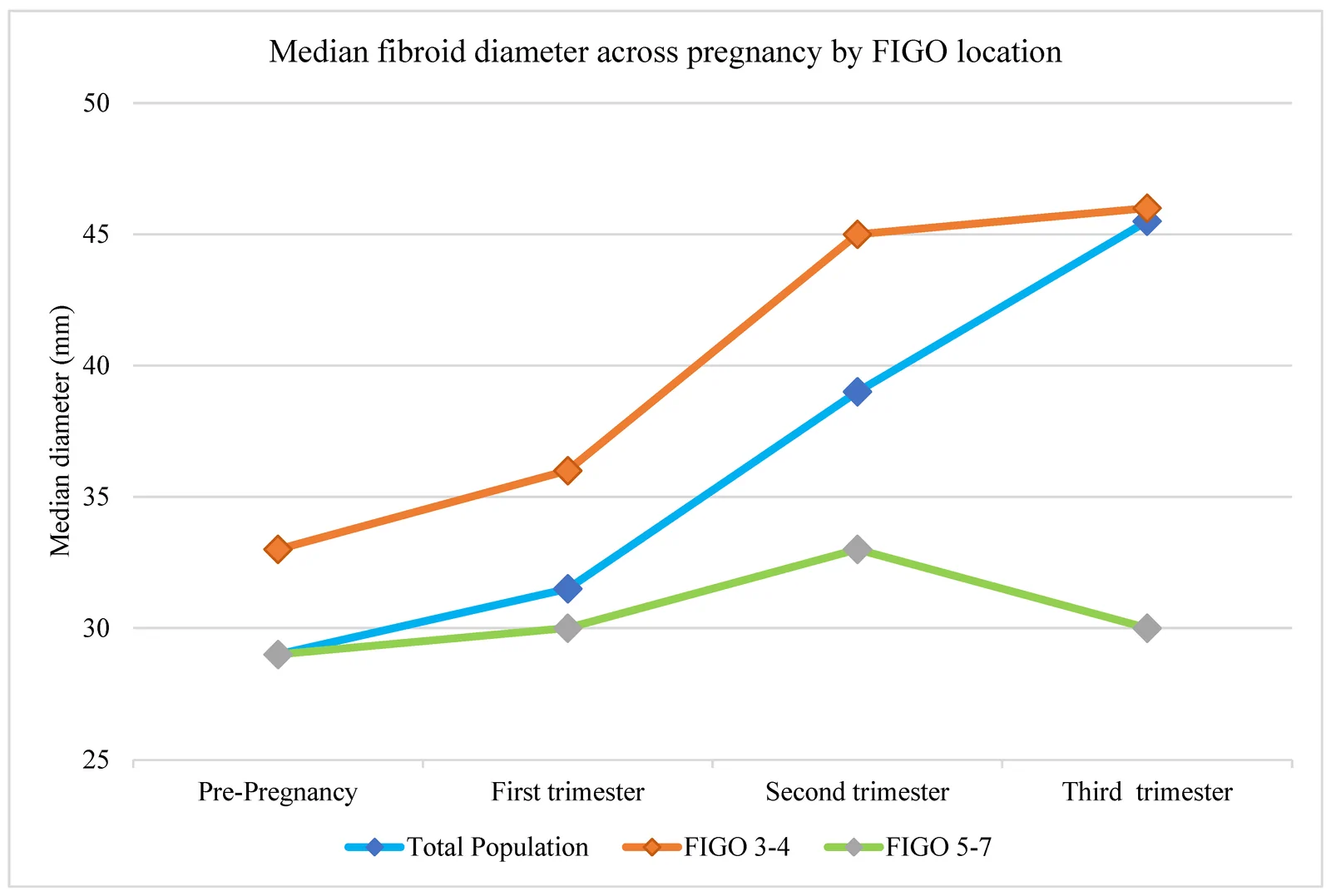
Longitudinal changes in uterine fibroid diameter from the preconceptional assessment (T0) through the first (T1), second (T2), and third (T3) trimesters of pregnancy. Lines represent median maximum fibroid diameter (mm) in the overall population with fibroids (n = 136), FIGO types 3–4 (n = 51), and FIGO types 5–7 (n = 85).

Regarding fibroid characteristics, 37.5% of lesions were classified as FIGO types 3–4 and 62.5% as FIGO types 5–7. Overall, fibroids showed a progressive increase in maximum diameter during pregnancy (*T*0 to *T*3). In linear mixed-effect models evaluating repeated measurements across timepoints, a significant main effect of time was observed (*p* < 0.001), with median diameter increasing from baseline (*T*0: 29 mm) through the first (*T*1: 31.5 mm), second (*T*2: 39.0 mm), and third trimesters (*T*3: 45.5 mm) (Figure 1); post hoc pairwise comparisons with Bonferroni’s correction confirmed significant growth between consecutive timepoints, particularly from *T*1 to *T*2 (*p* < 0.05).

When evaluating growth dynamics by FIGO classification, the LMM demonstrated a significant time-by-FIGO group interaction (*p* = 0.04). Specifically, FIGO type 3–4 fibroids exhibited a steady, progressive enlargement throughout gestation, reaching a larger median diameter in the second trimester compared to FIGO type 5–7 lesions (45.0 mm vs. 33.0 mm; post hoc *p* = 0.05), whereas FIGO type 5–7 fibroids remained relatively stable. Similarly, stratification by baseline size revealed a significant interaction (*p* = 0.03), with fibroids ≤5 cm at T0 demonstrating greater relative growth in maximum diameter by the second trimester compared with T0 than larger lesions (+18% vs. +7%; *p* = 0.03) despite maintaining smaller absolute dimensions.

## 4. Discussion

This retrospective cohort analyzed 1512 pregnant women, including 136 with uterine leiomyomas. Women with fibroids appear to have both a predisposing component related to family history and an acquired component related to hormonal exposure. In our cohort, women with fibroids more frequently had a family history of fibromatosis, supporting the role of genetic predisposition in leiomyoma development [24,25]. Consistently, maternal history has been associated with an increased risk of fibroid development, particularly when maternal fibroids are diagnosed at a younger age [26]. Consequently, pregnancy at an older maternal age exposes women not only to age-related risks but also to those associated with the presence of fibroids [7,27].

Similarly, women with fibroids had higher rates of smoking and alcohol consumption, thyroid disorders, chronic hypertension, and PMOS, and greater use of ART. This is consistent with the role of hormones in fibroid development; indeed, increased and/or prolonged exposure to hormonal stimulation increases the risk of developing fibroids and, consequently, of having them during pregnancy [5,6]. These associations may reflect shared endocrine, metabolic, inflammatory, or behavioral pathways relevant to fibroid biology and pregnancy-related complications [5,6,28]. Women with fibroids experienced menarche at an earlier age, supporting previous evidence identifying early menarche as an independent risk factor for leiomyoma development. Templeman et al. reported a 20–25% increased risk among women with menarche before 12 years of age [29]. This association may reflect prolonged cumulative exposure to estrogen and progesterone. Unexpectedly, no significant association was observed between endometriosis/adenomyosis and uterine fibroids. This finding should be interpreted cautiously because of the retrospective design, limited subgroup size, potential residual confounding, and, above all, the difficulty of diagnosing adenomyosis and endometriosis during pregnancy; diagnoses made before pregnancy would therefore be more reliable [30,31]. The differential diagnosis between adenomyosis and uterine fibroids may also be challenging outside pregnancy. Conway et al. described patients with adenomyosis who had initially been misdiagnosed with uterine fibroids, highlighting the clinical relevance of accurate ultrasound characterization when differentiating these myometrial disorders [32]. Moreover, adenomyosis is increasingly recognized as a heterogeneous condition whose reproductive and obstetric implications may vary according to disease phenotype and myometrial localization. Accurate phenotypic characterization may therefore be particularly relevant for reproductive counseling, pregnancy surveillance, and individualized management [33].

Furthermore, it would be useful to monitor women with congenital and acquired risk factors to provide appropriate counseling regarding their risk of developing fibroids with increasing age and to enable active surveillance for early detection.

Maternal age did not differ significantly between groups. Women with fibroids had a significantly lower pre-pregnancy BMI and lower gestational weight gain than controls. Although obesity has been recognized as a risk factor for fibroid development in the general population [34], our findings did not reproduce this pattern, highlighting the potential influence of population characteristics and residual confounding.

Beyond these established risk factors, modifiable lifestyle and nutritional exposures are increasingly being investigated in estrogen-dependent gynecological disorders. Dietary patterns may influence inflammatory and estrogen-related pathways in endometriosis, while recent evidence has similarly explored obesity and nutritional factors as potential modulators of fibroid development and growth [28,35]. These observations further support the multifactorial nature of leiomyoma development, in which genetic susceptibility interacts with hormonal, metabolic, and environmental factors.

Another important point concerns obstetric outcomes: beyond identifying risk factors for fibroid development and progression, it is equally important to determine whether their presence affects pregnancy and whether different fibroid sizes and locations are associated with different outcomes. Regarding obstetric outcomes, fibroids were associated with fewer vaginal deliveries, more operative vaginal and cesarean deliveries, and more second-degree tears among vaginal births. These findings are consistent with previous reports associating fibroids with an increased risk of cesarean delivery, potentially due to uterine distortion, impaired fetal descent, or altered myometrial contractility, particularly in cases involving large fibroids or lesions located in the uterine body or lower uterine segment [36]. Large fibroids (>5 cm) have also been associated with earlier delivery, preterm birth, and increased blood loss at delivery [37]. These considerations must therefore be taken into account when managing patients with fibroids to ensure appropriate care through referral to specialized care centers.

In contrast to previous literature reporting an increased risk of PPH in pregnancies complicated by fibroids [38], PPH was significantly less frequent among women with fibroids in our cohort. This unexpected finding should be interpreted cautiously and may reflect differences in obstetric management, closer surveillance, selection bias, or the relatively small number of patients with large or lower-segment fibroids.

Another factor to consider is neonatal outcomes, specifically whether newborns may be affected by the presence of fibroids. A higher prevalence of both FGR and LGA was observed in the fibroid group. Although these findings appear divergent, they may suggest that fibroids are associated with altered fetal growth patterns rather than with a uniform direction of growth impairment. Previous studies have more commonly described associations between fibroids and FGR, particularly in cases involving intramural or submucosal lesions close to placental implantation [39,40].

Regarding neonatal well-being, umbilical venous pH was significantly lower in the fibroid group; however, the absolute difference was small (7.31 vs. 7.32) and its clinical relevance should therefore be interpreted with caution. Umbilical cord blood gas parameters should not be interpreted in isolation, as biochemical differences may not necessarily correspond to clinically meaningful impairment in neonatal condition and should be considered together with other clinical indicators of neonatal adaptation [41].

Beyond conventional birth outcomes, maternal and placental pathology may also influence early neonatal adaptation and respiratory morbidity. In this context, a recent study of pregnancies complicated by placenta previa showed that histologically confirmed placenta accreta spectrum was associated with increased neonatal respiratory support requirements, including respiratory resuscitation at birth and supplemental oxygen need, even after adjustment for gestational age at delivery [42]. Although this clinical setting differs from pregnancies complicated by uterine fibroids, these findings highlight the importance of considering early neonatal adaptation and respiratory outcomes when evaluating the potential neonatal consequences of maternal and placental disease.

No significant differences were found in placental abnormalities, birth weight, gestational age at delivery, fetal malformations, or fetal sex distribution. These findings support the interpretation that fibroids may increase selected obstetric risks, particularly those related to delivery mode and fetal growth, without consistently worsening all immediate neonatal outcomes [36,37].

Multivariable analysis provided additional context to the univariable findings. After adjustment for maternal and pregnancy-related confounders, the association between uterine fibroids and cesarean delivery was attenuated and no longer statistically significant, while the inverse association with postpartum hemorrhage persisted. However, the latter finding should be interpreted cautiously given the small number of events and the possibility of residual confounding. The association with FGR was also attenuated after adjustment, with a wide confidence interval including the null value, whereas no meaningful association was observed with birthweight. The small reduction in umbilical venous pH remained statistically detectable, although its clinical relevance appears limited. Finally, the absence of preeclampsia events among women with fibroids precluded reliable adjusted estimation.

Stratification by FIGO classification showed differences in selected outcomes, including PPH, cesarean section, FGR, and LGA. FIGO type 3–4 fibroids were associated with higher rates of postpartum hemorrhage, cesarean section, and FGR, whereas FIGO type 5–7 fibroids were associated with higher rates of LGA and first-degree tears among women who delivered vaginally. These findings suggest that fibroid location may influence specific obstetric and neonatal outcomes, although interpretation should remain cautious because of the limited subgroup size and the potential contribution of fibroid size, number, and relationship with placental implantation [10].

Women with fibroids also showed a higher rate of conception through ART and a lower rate of spontaneous pregnancies. These findings are consistent with the work of Pritts et al., who identified submucosal fibroids as the subtype most strongly associated with impaired implantation and reduced clinical pregnancy rates, while surgical removal improved reproductive outcomes [43]. Intramural non-cavity-distorting fibroids may also negatively affect fertility through altered endometrial receptivity and impaired uterine perfusion. Generally, even when fibroid location may not affect a woman’s fertility, fibroid size and location should be considered when assessing risks in women using assisted reproductive technology (ART) for other conditions [43,44].

Pregnancy may also modify the clinical and sonographic behavior of other estrogen-dependent gynecological disorders. In endometriosis, lesions may undergo decidualization and changes in size and vascularity, and prospective ultrasound data indicate that many endometriomas and deep endometriotic nodules regress during gestation, although their course remains heterogeneous and pregnancy should not be regarded as a treatment for the disease [31,45]. Pregnancy-related hormonal and structural changes may also modify the sonographic appearance of adenomyosis [30]. These observations provide a useful biological framework for considering leiomyomas as dynamic lesions whose size and sonographic characteristics may likewise change throughout pregnancy and the puerperium.

Regarding fibroid evolution, our repeated-measure analysis confirmed significant time-dependent growth dynamics across gestation. Smaller fibroids showed greater relative growth during pregnancy, particularly during the second trimester, compared with larger lesions; however, this finding does not allow conclusions to be drawn regarding differential hormonal responsiveness. Furthermore, accounting for intra-individual trajectory differences revealed distinct growth patterns by anatomical location: FIGO type 3–4 fibroids exhibited progressive enlargement during the first two trimesters, whereas FIGO type 5–7 lesions remained relatively stable [46]. Increased intralesional vascularization was also observed in some cases, likely reflecting pregnancy-related hormonal and hemodynamic adaptations. Because fibroid size may change during pregnancy and the postpartum period, women planning subsequent pregnancies may benefit from postpartum reassessment and individualized preconception counseling [7,46]. At the same time, pregnancy-related enlargement or postpartum changes in fibroid size should not, by themselves, be interpreted as evidence of malignancy, as rapid growth alone is not considered a reliable predictor of uterine sarcoma [46,47]. Taken together, these findings support an individualized approach to pregnancies complicated by uterine fibroids. Serial ultrasound assessment may help evaluate interval changes in fibroid size and morphology, together with fetal growth and placental location, with surveillance tailored to fibroid size, location, growth pattern, maternal symptoms, and obstetric findings [7,48]. Particular attention may be warranted for large or enlarging lesions and for fibroids close to the endometrial cavity, placenta, cervix, or lower uterine segment, where their anatomical relationship may influence fetal growth or delivery planning. From a clinical perspective, uterine fibroids should therefore be regarded primarily as a factor that may increase the complexity of obstetric management rather than as a direct determinant of uniformly adverse pregnancy outcomes [7,10]. Management should encompass accurate early characterization, individualized antenatal surveillance, and appropriate delivery planning. In selected symptomatic cases or in women with large or anatomically relevant fibroids, preconception treatment may also be considered according to reproductive plans [13,44,49]. Finally, management should extend beyond delivery. Postpartum reassessment after uterine involution may help distinguish pregnancy-related changes from persistent fibroid enlargement and provide a more reliable baseline for counseling women planning subsequent pregnancies [7,44,46]. Persistent symptoms, cavity distortion, substantial residual fibroid size, or previous obstetric complications potentially related to fibroid characteristics should prompt individualized discussion of expectant management versus treatment before a subsequent conception.

Future multicenter prospective studies are needed to better define the relationship between fibroid characteristics and obstetric risk, evaluate the role of preconception treatment strategies, and develop more standardized management recommendations.

Several limitations should be considered. The stringent inclusion criteria—specifically requiring a documented preconceptional scan, serial trimester follow-ups, and institutional delivery—inherently selected a well-characterized tertiary-care cohort. While this design minimized missing longitudinal data and ensured standardized ultrasound evaluations, it may introduce selection bias towards patients with more severe symptoms, known fibroid pathology prior to pregnancy, or higher compliance, potentially limiting the generalizability of our findings to the broader obstetric population. The relatively small fibroid subgroup and the lower prevalence of uterine fibroids compared with published literature may have limited statistical power, particularly for rare outcomes [7]. Furthermore, the evaluation of multiple obstetric and neonatal outcomes and the performance of several subgroup comparisons increase the possibility of type I error; therefore, subgroup analyses according to FIGO classification and fibroid size should be considered exploratory and hypothesis-generating, particularly for findings based on nominal p values close to 0.05. Methodological heterogeneity may partly explain discrepancies with earlier retrospective studies and meta-analyses reporting increased risks of miscarriage, preterm birth, and cesarean delivery [36,50], whereas a large prospective community-based cohort did not confirm an increased risk of preterm birth associated with uterine fibroids [51]. Finally, neonatal outcomes were analyzed at the newborn level, and the potential within-pregnancy correlation between co-twins was not specifically modeled; however, twin pregnancies represented only 0.8% of the overall cohort. Additional limitations include residual confounding and the absence of postpartum follow-up, and potential inter-observer variability in ultrasound measurements. Although all sonographic examinations and fibroid measurements were performed by only two experienced maternal–fetal medicine specialists using the same ultrasound platform and a standardized assessment approach, some degree of measurement variability between operators cannot be entirely excluded. Nevertheless, this study provides clinically relevant information on fibroid behavior during pregnancy and supports risk stratification according to fibroid characteristics and maternal profile.

## 5. Conclusions

This study contributes additional evidence regarding the interaction between uterine fibroids and pregnancy, and fibroid changes during gestation. Although this condition requires careful obstetric monitoring, it does not appear to be consistently associated with adverse perinatal outcomes.

The mechanisms linking uterine fibroids to pregnancy complications are not yet fully understood, and further prospective studies are needed to better define them. In particular, a deeper understanding of the endocrine and vascular factors involved in the uteroplacental system and in embryo implantation may support the development of improved strategies for obstetric management in these patients.

Accordingly, management should extend from early ultrasound characterization and individualized antenatal follow-up to postpartum reassessment and, when appropriate, preconception treatment planning before subsequent pregnancies.

Overall, leiomyomas should be regarded as a condition requiring individualized obstetric surveillance rather than a contraindication to pregnancy.

## Figures and Tables

**Table 1:** Demographic and general clinical characteristics of the study population according to the presence of uterine fibroids.

PATIENTS’ CHARACTERISTICS	Total Population n = 1512	Patients with Uterine Fibroids n = 136	Patients Without Uterine Fibroids n = 1376	OR (95% IC)	*p* Value (Fibroids vs. No Fibroids)
**AGE (Years)**	35 ± 8.8	34 ± 8.9	35 ± 8.8	-	0.21
**CAUCASIAN** **ETHNICITY**	1300 (86.0%)	126 (92.6%)	1174 (85.3%)	2.17 (1.12–4.20)	**0.02**
**BODY MASS INDEX (Kg/m^2^)**	25.8 ± 5.0	24.4 ± 6.0	25.9 ± 5.0	-	**0.002**
**GESTATIONAL WEIGHT GAIN (Kg)**	15 ± 4.7	14 ± 3.4	15 ± 4.9	-	**0.02**
**SMOKING**	229 (15.1%)	36 (26.4%)	193 (14.0%)	2.21 (1.46–3.33)	**<0.001**
**ALCOHOL** **CONSUMPTION**	138 (9.1%)	28 (20.6%)	110 (8.0%)	2.98 (1.89–4.72)	**<0.001**
**THYROID** **DISORDERS**	134 (8.9%)	21 (15.4%)	113 (8.2%)	2.04 (1.23–3.38)	**0.01**
**TYPE I/II** **DIABETES** **MELLITUS**	22 (1.5%)	0 (0%)	22 (1.6%)	0.22 (0.01–3.66)	0.25
**CHRONIC** **HYPERTENSION**	23 (1.5%)	5 (3.7%)	18 (1.3%)	2.88 (1.05–7.88)	**0.04**

**Table 2:** Gynecologic and reproductive characteristics of the study population according to the presence of uterine fibroids.

GYNECOLOGIC AND REPRODUCTIVE CHARACTERISTICS	Total Population n = 1512	Patients with Uterine Fibroids n = 136	Patients Without Uterine Fibroids n = 1376	OR (95% IC)	*p* Value (Fibroids vs. No Fibroids)
**FIBROID FAMILY** **HISTORY**	324 (21.4%)	49 (36.0%)	275 (20%)	2.25 (1.55–3.28)	**<0.001**
**AGE AT** **MENARCHE**	12 ± 3.0	11 ± 1.0	13 ± 3.0	-	**<0.001**
**ENDOMETRIOSIS/ADENOMYOSIS**	58 (3.8%)	7 (5.1%)	51 (3.7%)	1.41 (0.63–3.17)	0.41
**POLYENDOCRINE METABOLIC OVARIAN SYNDROME** **(PMOS)**	92 (6.0%)	15 (11.0%)	77 (5.6%)	2.09 (1.17–3.75)	**0.01**
**NUMBER OF** **PREGNANCIES**	1 ± 1.3	1 ± 1.7	1 ± 1.3	-	1.0
**PARITY**	0 ± 0.8	0 ± 0.9	0 ± 0.8		1.0
**SPONTANEOUS PREGNANCY**	1382 (91.4%)	106 (77.9%)	1276 (92.7%)	0.27 (0.17–0.43)	**<0.001**
**ASSISTED** **REPRODUCTIVE TECHNOLOGIES (ART)**	130 (8.6%)	30 (22.1%)	100 (7.3%)	3.61 (2.29–5.68)	**<0.001**
**SINGLETON** **PREGNANCY**	1500 (99.2%)	133 (97.8%)	1367 (99.3%)	0.29 (0.08–1.09)	0.09
**TWIN PREGNANCY**	12 (0.8%)	3 (2.2%)	9 (0.7%)	3.43 (0.92–12.81)	0.09

**Table 3:** Obstetric outcomes according to the presence of uterine fibroids.

OBSTETRIC COMPLICATIONS	Total Population n = 1512	Patients with Uterine Fibroids n = 136	Patients Without Uterine Fibroids n = 1376	OR (95% IC)	*p* Value (Fibroids vs. No Fibroids)
**GESTATIONAL** **HYPERTENSION**	48 (3.2%)	3 (2.2%)	45 (3.3%)	0.67 (0.20–2.18)	0.63
**PREECLAMPSIA**	121 (8.0%)	0 (0%)	121 (8.8%)	0.04 (0.00–0.61)	**<0.01**
**ECLAMPSIA**	0 (0%)	0 (0%)	0 (0%)	-	1.0
**CHOLESTASIS**	14 (0.9%)	0 (0%)	14 (1.0%)	0.34 (0.02–5.80)	0.38
**GESTATIONAL** **HYPOTHYROIDISM**	112 (7.4%)	28 (20.6%)	84 (6.1%)	3.99 (2.49–6.38)	**<0.001**
**GESTATIONAL** **DIABETES**	233 (15.4%)	23 (17.0%)	210 (15.3%)	1.13 (0.71–1.81)	0.61
**PLACENTAL** **ABNORMALITIES**	13 (0.9%)	3 (2.2%)	10 (0.7%)	3.08 (0.84–11.33)	0.10
**THREATENED** **PRETERM LABOR**	3 (0.2%)	0 (0.0%)	3 (0.2%)	1.81 (0.07–27.98)	1.00
**POSTPARTUM** **HEMORRHAGE (PPH)**	172 (11.4%)	5 (3.7%)	167 (12.1%)	0.28 (0.11–0.68)	**0.003**
**LABOR** **ABNORMALITIES** **First stage > 12 h** **Second stage > 3–4 h**	3 (0.2%) 66 (4.4%)	0 (0%) 8 (5.9%)	3 (0.2%) 58 (4.2%)	1.44 (0.07–27.9) 1.42 (0.66–3.04)	1.00 0.37
**VAGINAL DELIVERY**	939 (62.1%)	64 (47.1%)	875 (63.6%)	0.51 (0.36–0.73)	**<0.001**
**VAGINAL TRAUMA** **First-degree tear** **Second-degree tear** **Third-degree tear** **Fourth-degree tear**	390 (38.4%) 312 (30.7%) 44 (4.3%) 0 (0%)	31 (40.3%) 33 (42.9%) 0 (0%) 0 (0%)	359 (38.3%) 279 (29.7%) 44 (4.7%) 0 (0%)	1.09 (0.68–1.75) 1.77 (1.10–2.84) - -	0.72 **0.02** 0.07 1.0
**OPERATIVE VAGINAL DELIVERY**	76 (5.0%)	13 (9.6%)	63 (4.6%)	2.20 (1.18–4.12)	**0.01**
**CESAREAN SECTION**	497 (32.9%)	59 (43.4%)	438 (31.8%)	1.64 (1.15–2.35)	**0.01**
**LABOR INDUCTION**	341 (22.6%)	26 (19.1%)	315 (22.9%)	0.80 (0.51–1.24)	0.32
**FGR**	58 (3.8%)	10 (7.4%)	48 (3.5%)	2.20 (1.08–4.45)	**0.03**
**SGA**	11 (0.7%)	0 (0%)	11 (0.8%)	0.43 (0.03–7.42)	0.62
**LGA**	72 (4.8%)	10 (7.4%)	62 (4.5%)	1.68 (0.84–3.36)	0.14

**Table 4:** Multivariable analyses of the association between uterine fibroids and obstetric and neonatal outcomes.

OUTCOME	Fibroids, n/N (%)	No Fibroids, n/N (%)	Adjusted Effect	95% CI	*p* Value
**CESAREAN DELIVERY**	59/136 (43.4)	438/1376 (31.8)	aOR 1.47 †	0.93–2.32	0.10
**POSPARTUM HEMORRAGHE**	5/136 (3.7)	167/1376 (12.1)	aOR 0.34 ‡	0.14–0.84	0.02
**PREECLAMPSIA**	0/136 (0)	121/1376 (8.8)	NE	—	—
**FGR**	10/136 (7.4)	48/1376 (3.5)	aOR 1.86 †	0.86–4.03	0.11
**BIRTHWEIGHT (g)**	3210 ± 443	3200 ± 523	β + 19.7 †	−73.5 to +112.8	0.68
**UMBILICAL VENOUS PH**	7.31 ± 0.05	7.32 ± 0.04	β − 0.011 †	−0.017 to −0.003	0.01

† Adjusted for maternal age, BMI, parity, ART, smoking, chronic hypertension, and diabetes mellitus/gestational diabetes mellitus. ‡ Firth’s penalized logistic regression because of the low number of events. Complete separation due to the absence of preeclampsia events in the fibroid group; the adjusted estimate is therefore not considered reliable.

**Table 5:** Fetal and neonatal outcomes according to the presence of uterine fibroids.

FETAL OUTCOME	Total Population n = 1524	Patients with Uterine Fibroids n = 139	Patients Without Uterine Fibroids n = 1385	OR (95% IC)	*p* Value (Fibroids vs. No Fibroids)
**FETAL** **MALFORMATIONS**	35 (2.3%)	2 (1.4%)	33 (2.4%)	0.60 (0.14–2.52)	0.75
**UMBILICAL** **VENOUS PH**	7.32 ± 0.04	7.31 ± 0.05	7.32 ± 0.04	-	**0.01**
**GESTATIONAL AGE**	39 ± 2.3	39 ± 1.7	39 ± 2.4	-	1.0
**BIRTH WEIGHT (g)**	3200 ± 518.7	3210 ± 443.3	3200 ± 522.5	-	0.83
**SEX MALE**	759 (49.8%)	66 (47.5%)	693 (50.1%)	0.90 (0.64–1.28)	0.57
**SEX FEMALE**	765 (50.2%)	73 (52.5%)	692 (49.9%)	1.11 (0.78–1.57)	0.57

**Table 6:** Obstetric complications observed in the fibroid population, stratified by FIGO groups (3–4 vs. 5–7).

OBSTETRIC COMPLICATIONS BY FIGO TYPE	Total Population with Fibroids n = 136	Patients with FIGO Type 3–4 Fibroids n = 51	Patients with FIGO Type 5–7 Fibroids n = 85	OR (95% IC)	*p* Value
**GESTATIONAL** **HYPERTENSION**	3 (2.2%)	3 (6%)	0 (0%)	-	0.05
**PREECLAMPSIA**	0 (0%)	0 (0%)	0 (0%)	-	1.0
**ECLAMPSIA**	0 (0%)	0 (0%)	0 (0%)	-	1.0
**CHOLESTASIS**	0 (0%)	0 (0%)	0 (0%)	-	1.0
**GESTATIONAL** **HYPOTHYROIDISM**	28 (20.6%)	10 (19.6%)	18 (21.2%)	-	0.83
**GESTATIONAL** **DIABETES**	23 (17.0%)	8 (15.7%)	15 (17.6%)	0.86 (0.34–2.21)	0.81
**PLACENTAL** **ABNORMALITIES**	3 (2.2%)	3 (5.9%)	0 (0%)	-	0.05
**THREATENED** **PRETERM LABOR**	0 (0.0%)	0 (0.0%)	0 (0.0%)	-	1.0
**POSTPARTUM** **HEMORRHAGE (PPH)**	5 (3.7%)	5 (9.9%)	0 (0.0%)	-	**0.01**
**LABOR** **ABNORMALITIES** **First stage > 12 h** **Second stage > 3–4 h**	0 (0%) 8 (5.9%)	0 (0%) 3 (5.9%)	0 (0%) 5 (5.9%)	- -	1.0 1.0
**VAGINAL DELIVERY**	64 (47.1%)	20 (39.2%)	44 (51.8%)	0.60 (0.30–1.22)	0.21
**VAGINAL TRAUMA** **First-degree tear** **Second-degree tear** **Third-degree tear** **Fourth-degree tear**	31 (40.3%) 33 (42.9%) 0 (0%) 0 (0%)	5 (21.7%) 13 (56.5%) 0 (0%) 0 (0%)	26 (48.1%) 20 (37.0%) 0 (0%) 0 (0%)	0.30 (0.10–0.92) 2.21 (0.82–5.96) - -	**0.04** 0.14 1.0 1.0
**OPERATIVE VAGINAL DELIVERY**	13 (9.6%)	3 (5.9%)	10 (11.8%)	0.47 (0.12–1.79)	0.37
**CESAREAN SECTION**	59 (43.4%)	28 (55.0%)	31 (36.5%)	2.12 (1.06–4.23)	**0.04**
**LABOR INDUCTION**	26 (19.1%)	10 (19.6%)	16 (18.8%)	1.05 (0.44–2.53)	0.91
**FGR**	10 (7.4%)	7 (13.7%)	3 (3.5%)	-	**0.03**
**SGA**	0 (0%)	0 (0%)	0 (0%)	-	1.0
**LGA**	10 (7.4%)	0 (0%)	10 (11.8%)	-	0.01

## Data Availability

The data that support the findings of this study are available from the corresponding author, E.C., upon reasonable request.

## Abbreviations

The following abbreviations are used in this manuscript:

ARTs	Assisted Reproductive Technologies
AUB	Abnormal Uterine Bleeding
BMI	Body Mass Index
FIGO	International Federation of Gynecology and Obstetrics
GDM	Gestational Diabetes Mellitus
FGR	Fetal Growth Restriction
LGA	Large for Gestational Age
MRI	Magnetic Resonance Imaging
PMOS	Polyendocrine Metabolic Ovarian Syndrome
PPH	Postpartum Hemorrhage
SGA	Small for Gestational Age
T0	Pre-pregnancy Ultrasound Assessment
T1	First-trimester Ultrasound Assessment
T2	Second-trimester Ultrasound Assessment
T3	Third-trimester Ultrasound Assessment
